# Predicting the effect of 5‐fluorouracil–based adjuvant chemotherapy on colorectal cancer recurrence: A model using gene expression profiles

**DOI:** 10.1002/cam4.2952

**Published:** 2020-03-09

**Authors:** Quan Chen, Peng Gao, Yongxi Song, Xuanzhang Huang, Qiong Xiao, Xiaowan Chen, Xinger Lv, Zhenning Wang

**Affiliations:** ^1^ Department of Surgical Oncology and General Surgery Key Laboratory of Precision Diagnosis and Treatment of Gastrointestinal Tumors Ministry of Education The First Affiliated Hospital of China Medical University Shenyang City China

**Keywords:** adjuvant chemotherapy, colorectal cancer, gene profile, machine learning

## Abstract

It is critical to identify patients with stage II and III colorectal cancer (CRC) who will benefit from adjuvant chemotherapy (ACT) after curative surgery, while the only use of clinical factors is insufficient to predict this beneficial effect. In this study, we performed genetic algorithm (GA) to select ACT candidate genes, and built a predictive model of support vector machine (SVM) using gene expression profiles from the Gene Expression Omnibus database. The model contained four ACT candidate genes (*EDEM1*, *MVD*, *SEMA5B*, and *WWP2*) and TNM stage (stage II or III). After using Subpopulation Treatment Effect Pattern Plot to determine the optimal cutoff value of predictive scores, the validated patients from The Cancer Genome Atlas database can be divided into the predictive ACT‐benefit/‐futile groups. Patients in the predictive ACT‐benefit group with 5‐fluorouracil (5‐Fu)–based ACT had significantly longer relapse‐free survival (RFS) compared to those without ACT (*P* = .015); However, the difference in RFS in the predictive ACT‐futile group was insignificant (*P* = .596). The multivariable analysis found that the predictive groups were significantly associated with the effect of ACT (*P*_interaction_ = .011). Consequently, we developed a predictive model based on the SVM and GA algorithm which was further validated to define patients who benefit from ACT on recurrence.

## Graphical abstract

We have developed an SVM‐GA model to predict the effect of 5‐fluorouracil (5‐Fu) based adjuvant chemotherapy(ACT) on recurrence in colorectal cancer (CRC) patients in this article. This model can help clinicians optimize their decision making for CRC patients who are suitable for 5‐Fu based ACT and avoid the adverse effect of chemotherapy on patients who are predicted to be ACT‐futile.

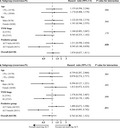

## 1 INTRODUCTION

Colorectal cancer (CRC) is the third most commonly diagnosed cancer and the leading cause of cancer‐related mortality in the world.^1^, ^2^ The treatment of 5‐fluorouracil (5‐Fu)–based adjuvant chemotherapy (ACT) following curative surgery is considered as the standard treatment for patients with stage II and III CRC who are at high risk of relapse.^3^ However, researchers have found that the rate of patients receiving ACT for stage II and III CRC is below 50%,^4^, ^5^ primarily due to the severe adverse effects of chemotherapy. In addition, there were some patients who have received ACT treatment experienced more harm effect than the good due to the significant adverse effects of chemotherapy that negatively affected their quality of life.^6^ Moreover, even after receiving the ACT treatment, the recurrence rate of stage II and III CRC in patients who received ACT is up to 30%.^7^ Therefore, to identify stage II and III CRC in patients who will benefit from adjuvant therapy has been defined as one of the most important areas in which to improve cancer patient care and outcomes.^6^

With evolution of high‐throughput technology, studies regarding the molecular mechanisms of disease and prognosis predictions for various cancers have made great progress by obtaining tumor genomic profiles.^8^ However, predictive models for the effect of ACT is found only in a limited number of studies 9 and were poorly in their predicting effects. Zheng et al^10^ has analyzed a sort of differentially expressed genes (DEGs) from stage II‐III drug‐resistant colorectal cell lines and developed a drug corresponding score system, while the DEGs from drug‐resistant colorectal cell lines may be irrelevant to drug sensitivity or resistance since they are simply supposed to identify the drug‐induced transcription changes.^11^ Additionally, Tong et al^12^ used relative expression orderings (REOs) and attained six gene pair‐based signatures (6‐GPSs) to predict the effect of ACT in patients with stage II‐III CRC; however, the reliability of this method has been questioned due to the limited robustness of independent data sets and differences among their outcomes.^9^ Furthermore, the results of previous models compared the survival differences among the patients who received 5‐Fu–based ACT, rather than identifying the patients who were suitable for 5‐Fu–based ACT.

It is difficult to directly build a predictive model using high‐dimensional profiles, since there are substantially more gene expression profiles than the number of presented samples, and still we need to conclude the clinical information and follow‐up information in analyzing matrix. A genetic algorithm (GA) has been reported to have the ability to efficiently select relevant features among massive gene expression values prior to model building.^13^ Also, the Support Vector Machine (SVM) is capable of recognizing subtle patterns in complex datasets, which is regarded as a supervised learning algorithm and is widely applied in analyzing classification of high‐dimensional data features.^14^ Moreover, some previous studies have successfully applied the SVM on the areas of cancer diagnosis and prognostic factors classification.^15^, ^16^

Therefore, we performed SVM with GA to select ACT candidate genes and build a predictive model, termed the SVM‐GA model, using the gene expression profiles from Gene Expression Omnibus (GEO) database. This predictive model was further validated using RNA sequence array expression profiles from The Cancer Genome Atlas (TCGA) database.

## 2 MATERIALS AND METHODS

### 2.1 Data sources and preprocessing

We downloaded the transcriptome profiling expression values of three cohorts as a training cohort (http://www.ncbi.nlm.nih.gov/geo/query/acc.cgi?acc=GSE14333, http://www.ncbi.nlm.nih.gov/geo/query/acc.cgi?acc=GSE29621, and http://www.ncbi.nlm.nih.gov/geo/query/acc.cgi?acc=GSE39582) from the GEO database (National Center for Biotechnology Information, US National Library of Medicine 8600 Rockville Pike, http://www.ncbi.nlm.nih.gov/geo/). These cohorts were collected from the same platform for cross‐cohort data comparison using a GPL570 [HG‐U133_Plus_2] Affymetrix Human Genome U133 Plus 2.0 Array. Meanwhile, we downloaded the level 3 mRNA sequence array expression data, fragments per kilobase million (FPKM), of patients with CRC from TCGA (https://cancergenome.nih.gov/) database portal as a test cohort. The detailed information of all cohorts was presented in Table 1.

**TABLE 1:** Datasets used in this study

Datasets	Type	Tissue	Platform	TNM Stage	ACT	Samples	Median follow‐up^c^
TCGA	mRNA	CRC tissue	HiSeqV2^a^	II‐III	5‐Fu based	138	72.53
http://www.ncbi.nlm.nih.gov/geo/query/acc.cgi?acc=GSE14333	mRNA	CRC tissue	GPL570^b^	II‐III	5‐Fu based	145	44.97
http://www.ncbi.nlm.nih.gov/geo/query/acc.cgi?acc=GSE29621	mRNA	CRC tissue	GPL570^b^	II‐III	5‐Fu based	31	52.86
http://www.ncbi.nlm.nih.gov/geo/query/acc.cgi?acc=GSE39582	mRNA	CRC tissue	GPL570^b^	II‐III	5‐Fu plus folinic acid	392	56.00

^a^ HiSeqV2 = IlluminaHiSeq_RNASeqV2.

^b^ GPL570 = GPL570[HG‐U133_Plus_2] Affymetrix Human Genome U133 Plus 2.0 Array.

^c^ Median follow‐up for relapse‐free survival time.

The cohorts selected for analyzing were based on the following criteria:

1. large‐scale human samples (numbers of patients > 29) of mRNA gene expression profiles were obtained from the untreated, primary cancer tissues;
2. measured using the same technology platform from which clinical information (including ACT, censored information, and TNM stage) and raw expression data are available;
3. the quality of data was evaluated or proven (accomplished via the peer review process or published in scientific journals).

All raw values were quantile normalized and transformed into a log_2_ scale base. Relapsed patients with relapse‐free survival (RFS) time below 36 months were moved to control bias. After deleting patients with unknown information, we included 706 patients with CRC in this study. Gene IDs in the microarray were mapped by probe IDs using the corresponding platform CDF files or using a DAVID Functional Annotation Tool (https://david.ncifcrf.gov/). If multiple probe IDs were mapped to the same gene IDs, the arithmetic average of the expression values of these probes were calculated. Probes that could not be mapped were removed. Furthermore, we prudentially deleted the genes for which the expression values were at 0 FPKM from at least 50% of the samples in the TCGA dataset. To reduce the experimental batch effect and unwanted deviation, we used ComBat to correct batches.^17^

### 2.2 Classifier for ACT benefit

To evaluate the appreciation of patients received ACT, we divided the patients into ACT‐benefit and ACT‐futile groups according to the treatment method of ACT and RFS time.^18^ The patients were subsequently classified based on: (a) ACT‐benefit group: patients whose RFS time was greater than 36 months who received ACT or was less than 36 months without ACT; (b) ACT‐futile group: patients whose RFS time was greater than 36 months treated without ACT or was less than 36 months received ACT.

### 2.3 Selection of ACT relevant genes and building a predictive model

After comparing the chi‐square values calculated using the Wilcoxon test, DEGs from the ACT‐benefit and ACT‐futile groups in the training cohort were selected to build a predictive model. Significant DEGs and clinical variables were used as SVM input and the ACT‐benefit/‐futile results were used as the outcome.

We used LIBSVM to build a model to predict the effect of ACT.^15^, ^19^ The kernel function is the radial basis function (RBF). The accuracy of the model outcomes is measured by calculating the area under the receiver operating characteristics curve (AUC). To increase the accuracy, some parameters (eg, cost(c) to reduce model overfitting and gamma (g) to control the degree of nonlinearity) were systematically optimized.

In this study, we performed GA by selecting variables according to previous studies.^15^, ^20^ The GA was based on the results of “evaluations” for all input “chromosomes” in the inputting variables in the training dataset and the most optimized variable subset was selected. In each generation, individuals were selected according to fitness, after which cross and mutation were constructed to a new set of solutions. The cardinal principle of GA is the process of natural selection; like natural evolution, after decoding the optimal individuals in the last generation can be used as an approximate solution to the problems.

To enhance the operating efficiency of the system algorithm and maximize the possibility of selecting best chromosome with the best fitness, we set the size of population, possibility of cross and possibility of mutation to 20, 10% and 30%, respectively. The iteration of GA was determined as 10 000 to reduce the possibility that the optimal solution by iteration of a single initial value. Fivefold cross‐validation (CV) was performed to reduce the bias of training samples over‐fit and helped to determine the best optimized parameters.^20^ Finally, the developed SVM‐GA model was used to calculate a predictive score for each patient. The source code of the SVM‐GA model was uploaded into the Github (https://github.com/QuanChen-cmu/SVM-GA-model).

### 2.4 Determination of model cutoff point

We used the Subpopulation Treatment Effect Pattern Plot (STEPP) to determine the cutoff points to classify the subgroup patients into predictive ACT‐benefit/‐futile groups.^21^ Using the STEPP, we plotted the changes during the 3‐year RFS time following the increasing predictive score, which was calculated by SVM‐GA model. According to the cutoff point determined by STEPP, patients in the test cohort would be stratified into two groups, and then a log rank test was performed to compare the difference in the RFS rate of patients with/without ACT between these two groups.

### 2.5 Functional enrichment analysis

We carried out a KEGG pathway analysis using R clusterProfiler^22^ and a Reactome pathway analysis (https://www.reactome.org/). A Fisher's exact test was used to select the relevant pathways.

### 2.6 Sensitivity analysis

Sensitivity analysis was performed using propensity score (PS) analysis in this part. The patients in test set were adjusted using the PS analysis by applying the nearest neighbor matching method. In this analysis, the PS match creates groups of patients with a similar probability of receiving the ACT on the basis of their baseline characteristics to minimize the differences among patients’ covariates, which could become confounding factors to evaluate the effect of ACT in a nonrandomized cohort.^23^, ^24^, ^25^ In this study, a PS of each patient means the likelihood of receiving ACT which was calculated by using a covariate adjustment method of clinicopathological factors. We initially performed logistic regression to select the significant clinicopathological factors which may effectively influence the evaluation of the effect of ACT. According to these covariates, a new set of unmatched patients was identified. By using a 1:1.5 nearest neighbor matching algorithm that pairs patients with the closest PS within a defined limit, the PS yielded two well‐matched patient sets (logistic analysis algorithm). After PS matching, we validated the model in defining patients that benefit from ACT upon recurrence using the newly matched patients set.

### 2.7 REO‐based signature

The REO‐based signature was promoted to predict the effect of 5‐Fu–based ACT for patients with stage II and III CRC.^12^, ^26^ We reobtained the CRC mRNA expression profiles of the test cohort and transformed them into a log2 scale. The duplicated genes were modified by calculating the arithmetic average of their expression. After comparing the expression orderings of the reported six gene pair signatures (6‐GPS), the patients with at least a half of the REOs of the set of gene pairs were stratified into the high‐risk group, whereas the residuals were stratified into the low‐risk group.

### 2.8 Statistical programs and software

A threshold value of *P* < .05 was considered statistically significant, except under special circumstances as described separately. All statistical analyses were performed using R version 3.5.3 (https://www.r-project.org/). The SVM algorithm was built using the LIBSVM program^27^ based on MATLAB 2016a (MathWorks), and the source code was uploaded to Github (https://github.com/QuanChen-cmu/SVM-GA-model). Meanwhile, the GA was coded based on MATLAB 2016a.

## 3 RESULTS

### 3.1 Data preprocessing and characteristics

**FIGURE 1:**
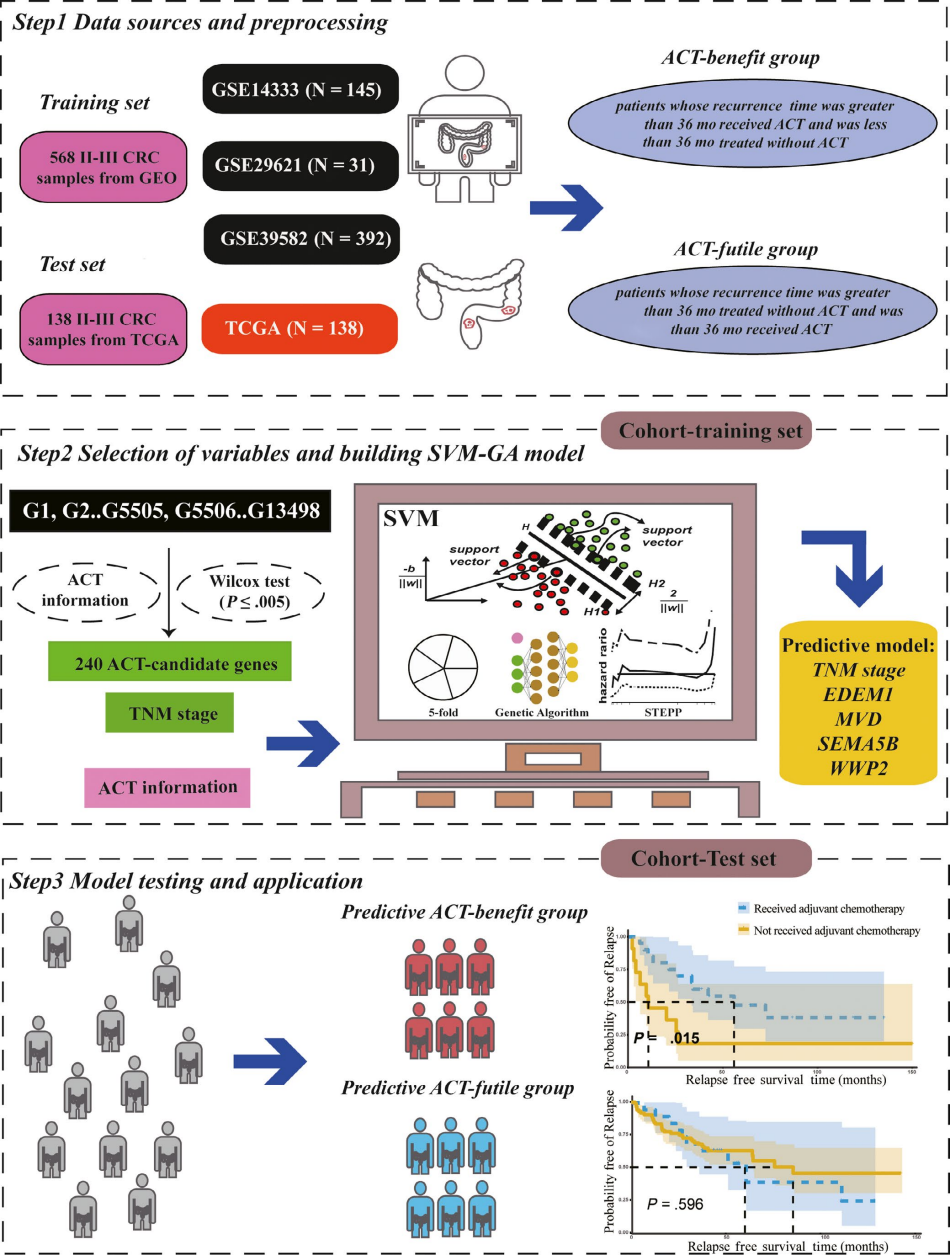
Outline of the SVM‐GA model flow

The outline of the overall study is shown in Figure 1. As is shown in Figure 1, the training cohort included 568 patients from GEO database and the test cohort included 138 patients from TCGA database. There were 401 (56.80%) patients with stage II CRC and 309 (43.77%) patients who received 5‐Fu–based ACT (Table S1). The clinicopathological factors in all datasets are presented in Table 2.

**TABLE 2:** Comparison of clinical factors between patients who received ACT and patients who did not receive ACT in all datasets

	Case number (N%)		*P* *
	Patients who received ACT	Patients who did not receive ACT	
Age^a^			<.001
<55	77 (26.64%)	45 (11.66%)	
55‐65	83 (28.72%)	62 (16.06%)	
65‐75	95 (32.88%)	136 (35.23%)	
>75	34 (11.67%)	143 (37.05%)	
Race^a^			.054
White	22 (55.00%)	56 (57.14%)	
Black/African	7 (17.50%)	4 (4.08%)	
Asian	1 (2.50%)	2 (2.05%)	
Unknown	10 (25.00%)	36 (36.73%)	
Gender			.290
Male	162 (52.42%)	224 (56.42%)	
Female	147 (47.58%)	173 (43.58%)	
Site			.184
Colon	292 (94.50%)	365 (91.94%)	
Rectum	17 (5.50%)	32 (8.06%)	
Grade^a b^			.787
I	1 (5.00%)	1 (9.09%)	
II	15 (75.00%)	7 (63.64%)	
III	4 (20.00%)	3 (27.27%)	
Histological type^a^			.709
AC	36 (90.00%)	86 (87.76%)	
MC	4 (10.00%)	12 (12.24%)	
T stage^a^			<.001
T1	4 (1.69%)	1 (0.33%)	
T2	37 (15.68%)	95 (31.05%)	
T3	160 (67.79%)	168 (54.89%)	
T4	35 (14.84%)	42 (13.73%)	
N stage^a^			<.001
N0	56 (24.03%)	161 (53.14%)	
N1	88 (37.77%)	111 (36.63%)	
N2	73 (31.33%)	22 (7.26%)	
N3	16 (6.87%)	9 (2.97%)	
TNM Stage			<.001
II	86 (27.84%)	315 (79.35%)	
III	223 (72.16%)	82 (20.65%)	

Abbreviations: AC, adenocarcinoma; Black/African, Black or African American; MC, mucinous adenocarcinoma.

^a^ Lack of information in some series: 31 patients, lack of age information (http://www.ncbi.nlm.nih.gov/geo/query/acc.cgi?acc=GSE29621); 164 patients, lack of T stage information (http://www.ncbi.nlm.nih.gov/geo/query/acc.cgi?acc=GSE14333, http://www.ncbi.nlm.nih.gov/geo/query/acc.cgi?acc=GSE29621); 170 patients, lack of N stage information (http://www.ncbi.nlm.nih.gov/geo/query/acc.cgi?acc=GSE14333, http://www.ncbi.nlm.nih.gov/geo/query/acc.cgi?acc=GSE29621, http://www.ncbi.nlm.nih.gov/geo/query/acc.cgi?acc=GSE39582); Grade information was only provided in http://www.ncbi.nlm.nih.gov/geo/query/acc.cgi?acc=GSE29621 series; histological type and race information were only provided in TCGA series.

^b^ Grade I = well differentiated; grade II = moderately differentiated; grade III = poorly differentiated.

* *P* values were made by *χ* ^2^‐test.

### 3.2 Selection of 5‐FU–based ACT candidate genes and building the SVM‐GA model

After performing a Wilcoxon test on the expression values of genes in the training cohort between patients in the ACT‐benefit group and ACT‐futile group, we identified 240 significant DEGs (*P* < .001 as the threshold; Supplementary file1).

**FIGURE 2:**
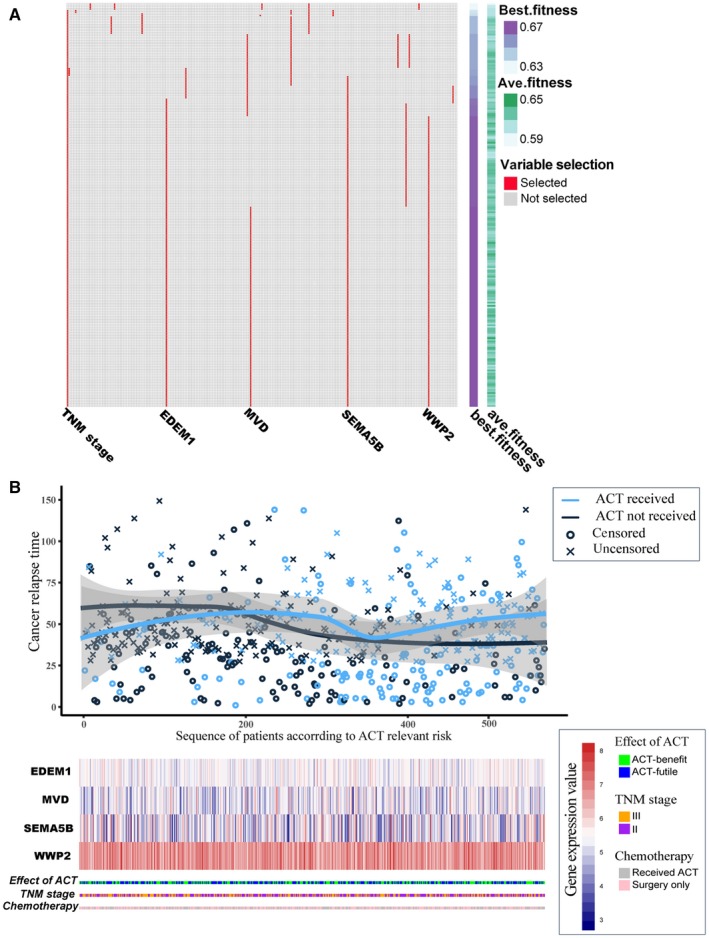
Establishment of the SVM‐GA model using the training cohort. A, Results of genetic algorithm (GA). The iterations of each variable in GA are presented in the longitudinal axes and the selected variables in the SVM‐GA model are presented in the transverse axes. B, SVM‐GA model predictive scores distribution, patient relapse‐free survival time, and expression heatmap

With the help of SVM and GA, we constructed and optimized a predictive model by setting the TNM stage (stage II or III); 240 ACT candidate genes as the input variables and the information for the ACT‐benefit/‐futile groups as the outcome. The average fitness and the best fitness of each iteration increased progressively and was sustained at a steady level in the process of GA iteration evolution (Figure 2A). Finally, we obtained a model containing four genes and TNM stage (stage II or III) after parameter optimization (training dataset AUC = 0.703). The best optimized genes’ combination was *EDEM1*, *MVD*, *SEMA5B*, and *WWP2*. The model calculated a predictive score for each patient and that patients who received 5‐Fu–based ACT in the training cohort had a longer RFS with the increase in predictive scores, whereas those without 5‐Fu–based ACT exhibited a slightly downward RFS trend (Figure 2B).

### 3.3 Determining the cutoff point of the SVM‐GA model

**FIGURE 3:**
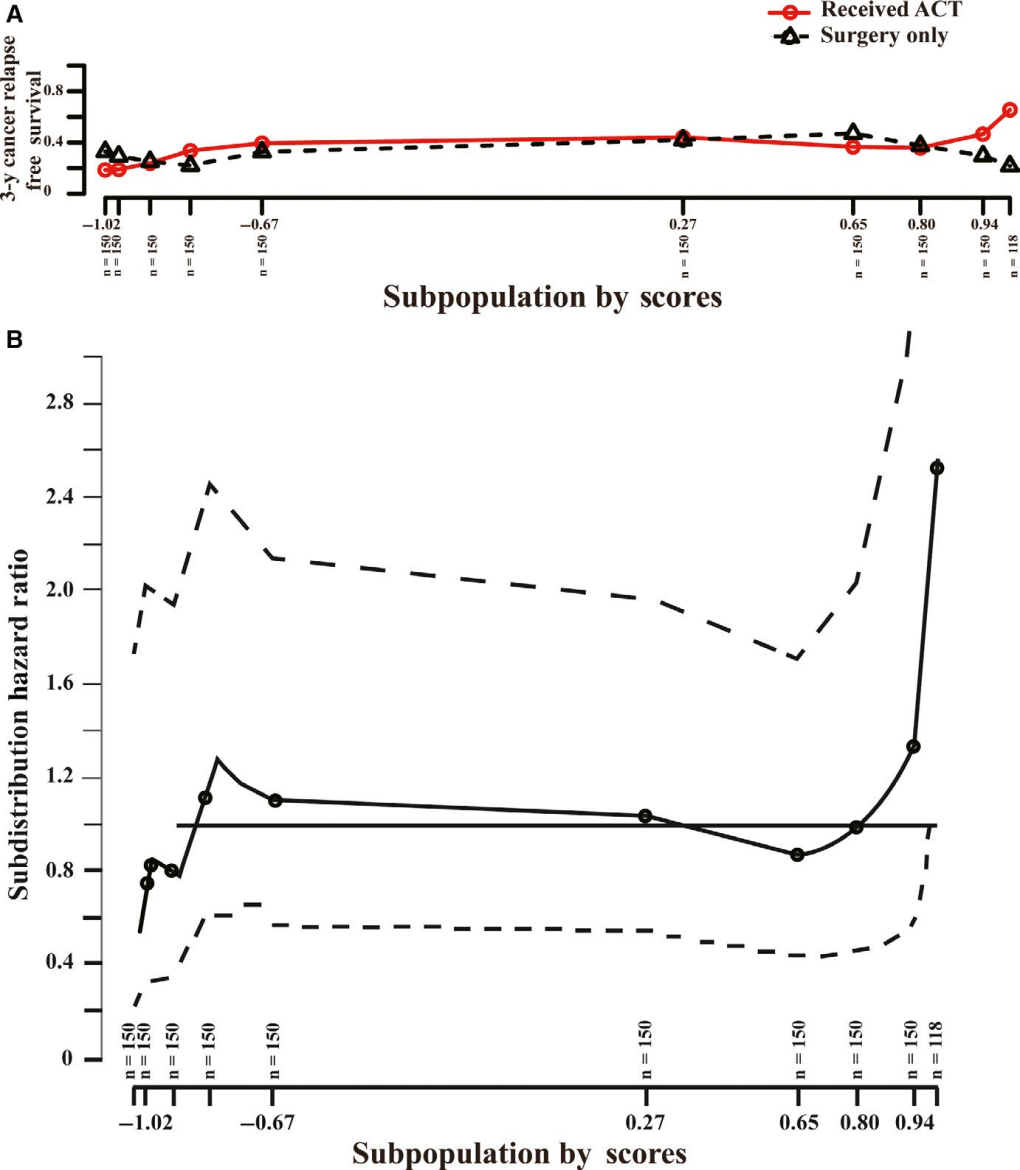
STEPP analysis between the concentrated continuous variables (predicted values of SVM model) and the effect of adjuvant chemotherapy (ACT) in the training cohort. A, Relapse‐free survival (RFS) rates at 36 mo of patients with ACT and surgery only according to patients’ subpopulations clustered by predictive values. B, Hazard ratio of patients with ACT according to patients’ subpopulations clustered by predictive values (solid line) with a 95% confidence interval (dashed lines)

To classify patients into the predictive ACT‐benefit/‐futile groups, we used the STEPP to determine the cutoff point for the predictive scores. The results showed that there was a significant tendency toward ACT and both 3‐year RFS differences (Figure 3A) and hazard ratio (Figure 3B) following the increasing predictive scores. Indeed, the patients who received ACT with a higher predictive score (the predictive scores greater than 0.8) tended to have a longer RFS compared to those with surgery only. Therefore, we grouped the patients into the predictive ACT‐benefit group if the cutoff point greater than 0.8, and the remaining patients were grouped into the predictive ACT‐futile group. For the predictive ACT‐benefit group in training cohort, patients with surgery only had a significantly shorter RFS (*P* = .012, HR = 0.528, 95%CI = 0.318‐0.876; Figure S1A). There were no significant differences between the patients who received 5‐Fu–based ACT and those with surgery only in the predictive ACT‐futile group (*P* = .059, HR = 1.308, 95%CI = 0.989‐1.729; Figure S1B).

### 3.4 Validation of the SVM‐GA model

**FIGURE 4:**
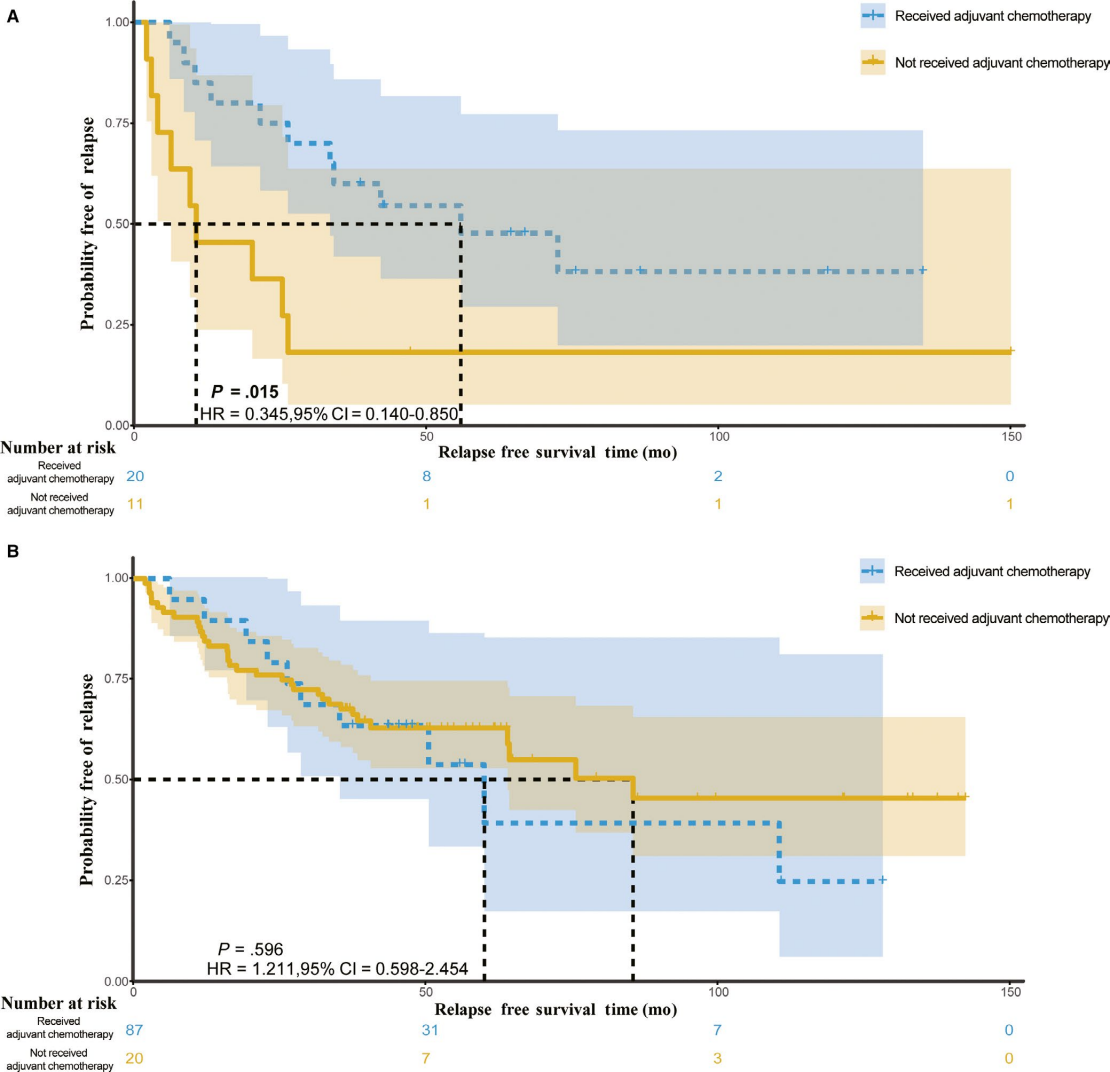
Relapse‐free survival (RFS) in the predictive adjuvant chemotherapy (ACT) groups in the test cohort. In total, 138 patients with CRC from TCGA database are included in these analyses. A, RFS in the predictive ACT‐benefit group. B, RFS in the predictive ACT‐futile group. 95%CI, 95% confidence interval; HR, hazard ratio

We validated the predictive signatures in the test cohort from the TCGA dataset. Based on the determined cutoff point, the SVM‐GA model stratified 138 patients into a predictive ACT‐benefit group that included 31 (22.46%) patients and a predictive ACT‐futile group that included 107 (77.54%) patients. The patients who received 5‐Fu–based ACT in the predictive ACT‐benefit group had a significantly longer RFS than those with surgery only (*P* = .015, HR = 0.345, 95%CI = 0.140‐0.850; Figure 4A); however, there was no significant difference between these two types in the predictive ACT‐futile group (*P* = .596, HR = 1.211, 95%CI = 0.598‐2.454; Figure 4B). Therefore, the results of external validation suggested that this predictive model could distinguish between patients who were and were not suitable for receiving ACT.

**FIGURE 5:**
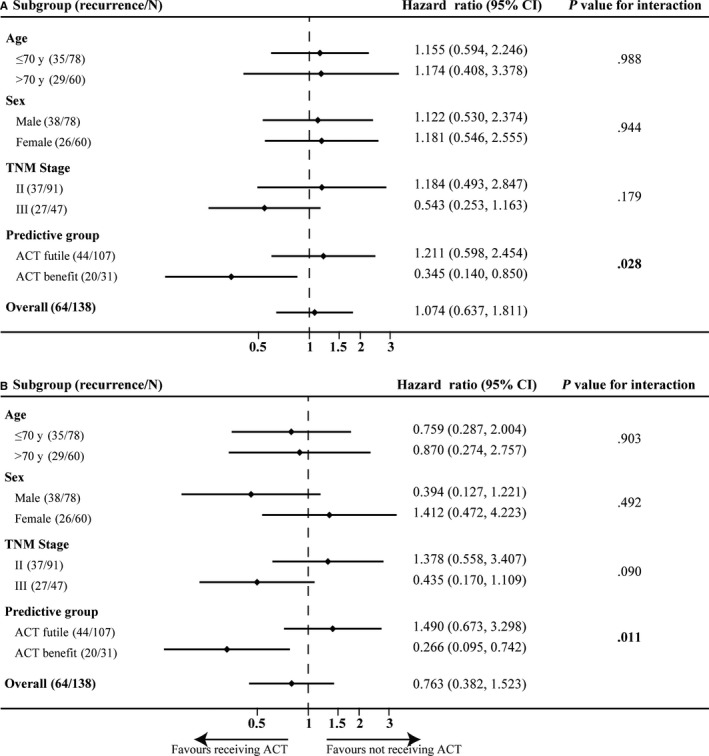
Association between the relapse‐free survival (RFS) and predictive adjuvant chemotherapy (ACT) groups or clinical characteristics in the test cohort. A, Univariate analysis. B, Multivariate analysis adjusted for age, sex, TNM stage, and predictive groups. *P* values for association between clinical characteristics and ACT‐benefit group

Furthermore, we performed univariate and multivariate regression analysis to identify the association between the effect of ACT and clinical characteristics. Among the patients in the predictive ACT‐benefit group, those with ACT were significantly associated with a longer RFS compared to those with surgery only (univariable analysis HR = 0.345, 95%CI = 0.140‐0.850, *P* = .021; multivariable analysis HR = 0.266, 95%CI = 0.095‐0.742, *P* = .011; Figure 5, Table S2). In contrast, the results between the patients who received 5‐Fu–based ACT and those received surgery only in the predictive ACT‐futile group was not significant (univariable analysis HR = 1.211, 95%CI = 0.598‐2.454, *P* = .595; multivariable analysis HR = 1.490, 95%CI = 0.673‐3.298, *P* = .325; Figure 5, Table S2). The associations on RFS between ACT and the predictive ACT groups (ACT‐benefit group vs ACT‐futile group) regarding RFS were significant (univariable analysis *P*_interaction_ = .028; multivariable analysis *P*_interaction_ = .011; Figure 5). However, there was no significant association on RFS between ACT and the other characteristics (Figure 5).

### 3.5 Sensitivity analysis

**FIGURE 6:**
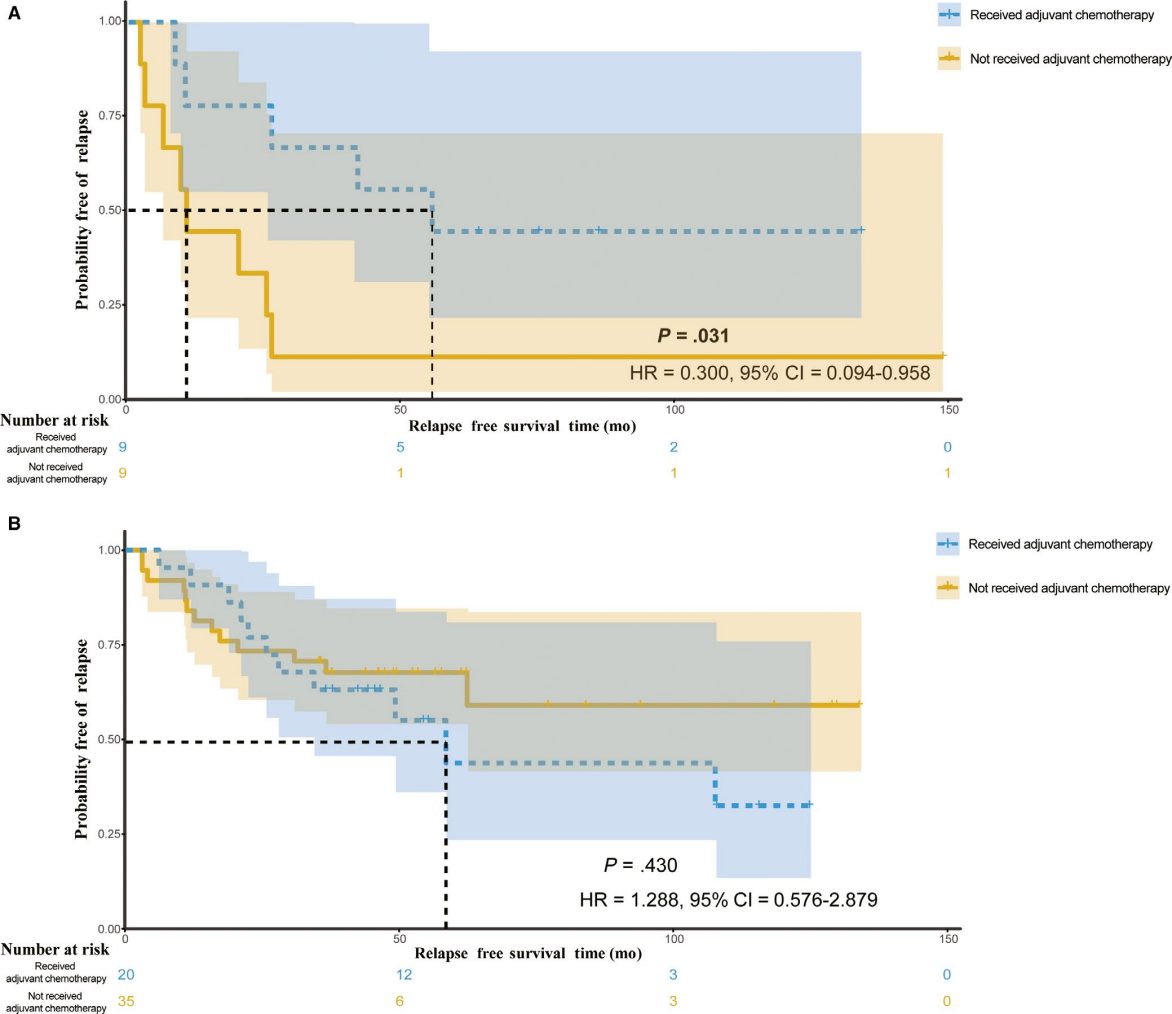
Relapse‐free survival (RFS) in the predictive adjuvant chemotherapy (ACT) groups after PS matching in the test cohort. In total, 73 patients with CRC from TCGA database are included in these analyses. A, RFS in the predictive ACT‐benefit group. B, RFS in the predictive ACT‐futile group. 95%CI, 95% confidence interval; HR, hazard ratio

Before PS matching, there were five factors (age, sex, T stage, N stage, and TNM stage) can significantly influence the effect of ACT by univariate logistic regression (Table S3). We obtained seventy‐three patients in the newly test set after PS matching, the differences of clinicopathological factors were insignificant between the patients who received adjuvant chemotherapy and those who did not (Table 3). In sensitivity analyses, patients who received ACT in the predictive ACT‐benefit group remained significantly longer RFS than those who did not received ACT (*P* = .031, HR = 0.300, 95%CI = 0.094‐0.958; Figure 6A). Additionally, there was no significant difference between these two types in the predictive ACT‐futile group (*P* = .430, HR = 1.288, 95%CI = 0.576‐2.879; Figure 6B).

**TABLE 3:** Baseline characteristics before and after propensity score analysis in the test cohort

Characteristic	Before matching			After matching		
	Patients who received ACT (N = 40)	Patients who did not receive ACT (N = 98)	*P* *	Patients who received ACT (N = 29)	Patients who did not receive ACT (N = 44)	*P* *
Age			<.001			.072
<55	14 (35.00%)	12 (12.24%)		5 (17.24%)	11 (24.99%)	
55‐65	12 (30.00%)	16 (16.33%)		11 (37.93%)	7 (15.91%)	
65‐75	11 (27.50%)	38 (38.78%)		10 (34.48%)	13 (29.55%)	
>75	3 (7.50%)	32 (32.65%)		3 (10.35%)	13 (29.55%)	
Gender			.004			.669
Male	15 (37.50%)	63 (64.28%)		14 (48.27%)	19 (43.18%)	
Female	25 (62.50%)	35 (35.72%)		15 (51.73%)	25 (56.82%)	
T stage			.019			.342
T1	2 (5.00%)	1 (1.02%)		1 (3.45%)	1 (2.27%)	
T2	31 (77.50%)	92 (93.88%)		22 (75.86%)	39 (88.64%)	
T3	7 (17.50%)	5 (5.10%)		6 (20.69%)	4 (9.09%)	
N stage			<.001			.197
N1	11 (27.50%)	80 (81.64%)		11 (37.93%)	26 (59.10%)	
N2	17 (42.50%)	9 (9.18%)		10 (34.48%)	9 (20.45%)	
N3	12 (30.00%)	9 (9.18%)		8 (27.59%)	9 (20.45%)	
TNM stage			<.001			.077
II	11 (27.50%)	80 (81.64%)		11 (37.93%)	26 (59.09%)	
III	29 (72.50%)	18 (18.36%)		18 (62.07%)	18 (40.91%)	

* *P* values were made by *χ* ^2^‐test.

### 3.6 Evaluation of the SVM‐GA model stratified by TNM stage subgroups

We stratified patients in the predictive groups using the TNM stage and found that neither stage II nor III patients in the predictive ACT‐futile group exhibited a significant difference between the patients who received ACT and those with surgery only (*P* = .707 and *P* = .896 for stage II and III patients, respectively; Figure S2). There were no patients with stage II stratified into the predictive 5‐Fu–based ACT‐benefit group (Supplementary file2). The selected patients with stage III received ACT in the predictive ACT‐group had a significantly longer RFS compared with those with surgery only, while this was consistent with the previous finding (*P* = .015; Figure 5A).

### 3.7 Functional analysis on ACT‐relevant genes

The top 10 significant pathways according to KEGG and Reactome are presented in Figure S3 (Fisher's exact test, *P* < .05). The DEGs with high expression values in the ACT‐benefit group were mostly enriched in the pathways relevant to *MAPK*, *NTRs*, and *Notch* (Figure S3A,B), whereas genes with high expression values in the ACT‐futile group were mostly enriched in the pathways about Nonsense‐Mediated Decay (*NMD*) and *p53* signaling (Figure S3C,D).

### 3.8 Evaluation the effectiveness of the 6‐GPS REO‐based signature

We compared the relative orderings of 6‐GPS and stratified the patients into 5‐Fu–based high‐/low‐risk groups (Figure S4A). In both the predictive 5‐Fu–based high‐ and low‐risk groups, there were no significant RFS differences between the patients who received ACT and those with surgery only (*P* = .676 for high‐risk group and *P* = .222 for low‐risk group; Figure S4B,C). Similarly, there were also no significant RFS differences between high‐ and low‐risk group among patients who received ACT or those with surgery only (*P* = .113 for patients who received ACT and *P* = .818 for patients with surgery only; Figure S4D,E). Therefore, the 6‐GPS REO‐based signature was not considered suitable for the test cohort.

## 4 DISCUSSION

In 2019, the American Society of Clinical Oncology proposed that defining patients who would benefit from adjuvant therapy is a secondary priority area for accelerating progress against cancer and improving patient therapy outcomes.^6^ However, there remains an absence of proof with regard to which biomarkers can be used to predict the effect of 5‐Fu–based ACT on recurrence for stage II‐III CRC patients. Moreover, some studies^10^, ^12^ developed models to predict the effect of ACT on prognosis using gene expression profiles with unsatisfactory results. Zheng et al^10^ built a drug corresponding score system using DEGs from stage II‐III drug‐resistant colorectal cell lines, in which the DEGs were proposed to identify drug‐induced transcriptional changes rather than drug sensitivity or resistance.^11^ Therefore, we thought that the predictive model based on DEGs from drug‐resistant colorectal cell lines was not suitable for predicting the effect of ACT. Additionally, Tong et al 12 developed a REO‐based signature to predict the effect of 5‐Fu–based ACT. However, when we processed the 6‐GPS in the test cohort, no significant difference was found between the patients who received ACT and those with surgery only in both the high‐ and low‐risk groups (Figure S4B,C). This unwanted result was partially due to that REO was a rank‐based model and some subtle quantitative information might be lost.^28^ Meanwhile, the 6‐GPS was presented by comparing the differences in survival among patients who received ACT rather than directly distinguishing patients who were suitable for 5‐Fu–based ACT.

In this study, we built a predictive model to define the patients who will be suitable for 5‐Fu–based ACT, termed the SVM‐GA model. The model was built based on the SVM algorithm which was a robust algorithm that could select the most optimized subset with the help of a GA. Based on the external validation, patients who received ACT in the predictive ACT‐benefit group had a longer RFS than those with surgery only (*P* = .015; Figure 4A), whereas there was no significant difference between two patient subsets in the predictive ACT‐futile group (*P* = .596; Figure 4B). This demonstrated that the predictive model can directly distinguish between patients who were and were not suitable for ACT. The SVM‐GA model can be further validated using the uploaded code. At the same time, in order to evaluate the robustness of the SVM‐GA model, we performed a multivariable analysis. Except our model, there was no significant association between the effect of 5‐Fu–based ACT and the variables containing the TNM stage, age, and sex (*P*_interaction_ > .05; Figure 5). Thus, the SVM‐GA model can be applied in clinical decision using the code we uploaded to directly distinguish the patients who would be ACT‐benefit or not.

We directly compared the ACT‐received subgroups of the ACT‐benefit and ACT‐futile groups and found the result was not significant (median RFS of ACT‐received patients: 60.033 months vs 55.933 months for the predictive ACT‐futile and ACT‐benefit groups, respectively; log rank *P* = .845). The main function of our SVM‐GA model may be that it could tell the CRC patients whether they need ACT if a certain series of genes were expressed on primary tumor. We suggested the predictively ACT‐benefit patients should receive ACT as possible, otherwise the patients’ RFS will be significantly shortened (3‐year relapse rate: 18.2% vs 60.0% for the patients without ACT and the patients with ACT, respectively; *P* = .015; Figure 4A). Additionally, as for as the patients were predicted with ACT‐futile, the tendency to relapse after surgery would not be significant if they refused ACT for some reasons (3‐year relapse rate: 69.0% vs 65.0% for the patients without ACT and the patients with ACT, respectively; log rank *P* = .596; Figure 4B).

Since the TNM stage of CRC in patients is considered to be one of the independent factors that can impact the effect of 5‐Fu–based ACT,^29^, ^30^ we performed a subgroup analysis to identify the influence of the TNM stage in this study. The results suggested that our model was able to distinguish the patients with stage III who should be suitable for ACT (*P* = .015; Figure 4A). However, interestingly, patients with stage II CRC was completely predicted to be ACT futile. Some recent studies have reported there were no significant effect of ACT on both DFS and OS between patients with stage II CRC who received 5‐Fu–based ACT and those with surgery only.^31^, ^32^ After analyzing the patients with stage II in the test cohort, we found there was no significant association between the patients who received ACT and those with surgery only (HR = 1.183, 95%CI = 0.492‐2.844, *P* = .707; Figure S2A). Because the patients with stage II CRC in the test cohort actually had worse RFS compared to those with surgery only, we cannot come to conclusion that the SVM‐GA model was not able to accurately define the stage II CRC patients who will benefit from ACT. Simultaneously, this research sample size in the test cohort was small. Besides, our predictive model should be expected to distinguish the stage II CRC patients who will benefit from 5‐Fu–based ACT after expanding the number of study patients.

Four genes (*EDEM1*, *MVD*, *SEMA5B*, and *WWP2*) and TNM stage (stage II or III) were included when establishing the SVM‐GA model. We also performed KEGG and Reactome analyses based on the selected genes with high expression values in the ACT‐benefit/‐futile groups. Except the *EDEM1*, the expression levels of the other genes in the ACT‐benefit group were upregulated. The downregulation of *EDEM1* has been reported to be correlated with a strong activation of cellular autophagy,^33^ which could improve sensitivity to chemotherapy and promote the death of tumor cells.^34^ The function of *MVD* is to mediate the relative expression of protein kinase B (*Akt*)^35^, ^36^; the level of *Akt* has also been reported to decrease the resistance to 5‐Fu in CRC cells possibly by activating the *PI3K/AKT* pathway.^37^ The enrichment results of ACT‐benefit genes using Reactome suggested that the beneficial effect of ACT was strongly associated with the “*PI5P*, *PP2A*, and *IER3* Regulate *PI3K/AKT* Signaling” pathway (Figure S3A,C), as these regulators could activate *PI3K/AKT* signaling and inhibit *Akt* dephosphorylation to overcome 5‐Fu resistance in CRC cells.^38^ *WWP2* has been reported to improve the sensitivity to chemotherapy^39^ by binding to *Notch3* in ovarian cancer cells and inducing *WWP2* associated *Myc* degradation in myeloma cells.^40^, ^41^ Moreover, a functional analysis found *NMD* pathways correlated with the futile effect of ACT. Furthermore, researchers identified CRC cells with* NMD* activity to be correlated to microsatellite sequence instability (MSI)^42^; CRC cells with MSI were found to become more resistant to 5‐Fu than those with microsatellite sequence stability (MSS).^43^, ^44^ However, while the relationship between *SEMA5B* and chemotherapy in CRC is unclear, it has been shown to activate both calcineurin and calpain‐mediated pathways,^45^ which could functionally enhance tumor cell autophagy and apoptosis.^46^ Although we identified some relevant ACT signatures, evidence regarding the specific molecular mechanism of these signatures remains unclear which required a further experimental validation. Overall, our model used these signatures to determine the optimal chemotherapy options for patients with stage II‐III CRC.

This study had some limitations. The cutoff point was determined using normalized profiles; hence, a large‐scale sample is required to validate this best cutoff point, which can be measured by real‐time PCR or assays using paraffin‐embedded specimens as a standard. In addition, these public datasets were lacking additional clinical information (eg, the number of dissected lymph nodes, MSI and histological type), which is necessary to define high‐risk stage II CRC patients and validate the robustness of our predictive model. Also, there were also lacking information on chemotherapy's poisonous side effects, which should be considered when making a proper recommendation on ACT to a given patient. Moreover, it can be expected that the candidate four genes deduced from transcriptional abundance could be measured by some experimental methods such as reverse transcriptase PCR (RT‐PCR) or in situ hybridization in primary CRC tissues. Thus, it is worth developing biological confirmation to measure the four candidate genes for the clinical application of the SVM‐GA model in future prospective studies.

In summary, we developed an SVM‐GA model to predict the effect of 5‐Fu–based ACT on recurrence in patients with CRC. This model can help clinicians optimize their decision making for patients with CRC who are suitable for 5‐Fu–based ACT and avoid the adverse effect of chemotherapy on patients who are predicted to be ACT‐futile. However, further studies are needed to validate these results.

## CONFLICT OF INTEREST

The authors declare that they have no conflicts of interest.

## AUTHOR CONTRIBUTION

ZNW, PG, and QC conceived, designed, and supervised the study. QX and XL collected the expression data. PG, YXS, and QC developed the SVM‐GA model and provided the code. QC did the statistical analysis. QC, XZH, and XWC wrote and revised the manuscript. All authors had full access to the data, discussed and reviewed the manuscript, and approved the manuscript for publication.

## Supporting information

FigS1

FigS2

FigS3

FigS4

TableS1‐S3

Supinfo1

Supinfo2

## Data Availability

The datasets generated and analyzed during the current study are available in the Gene Expression Omnibus (GEO) database (http://www.ncbi.nlm.nih.gov/geo/) and The Cancer Genome Atlas (TCGA) database (https://cancergenome.nih.gov/).
