# Supplementary figures and images for: Predicting the effect of 5‐fluorouracil–based adjuvant chemotherapy on colorectal cancer recurrence: A model using gene expression profiles
Source: Cancer Med. 2020 Mar 9;9(9):3043–56. doi: 10.1002/cam4.2952 (PMC7196071; doi:10.1002/cam4.2952)

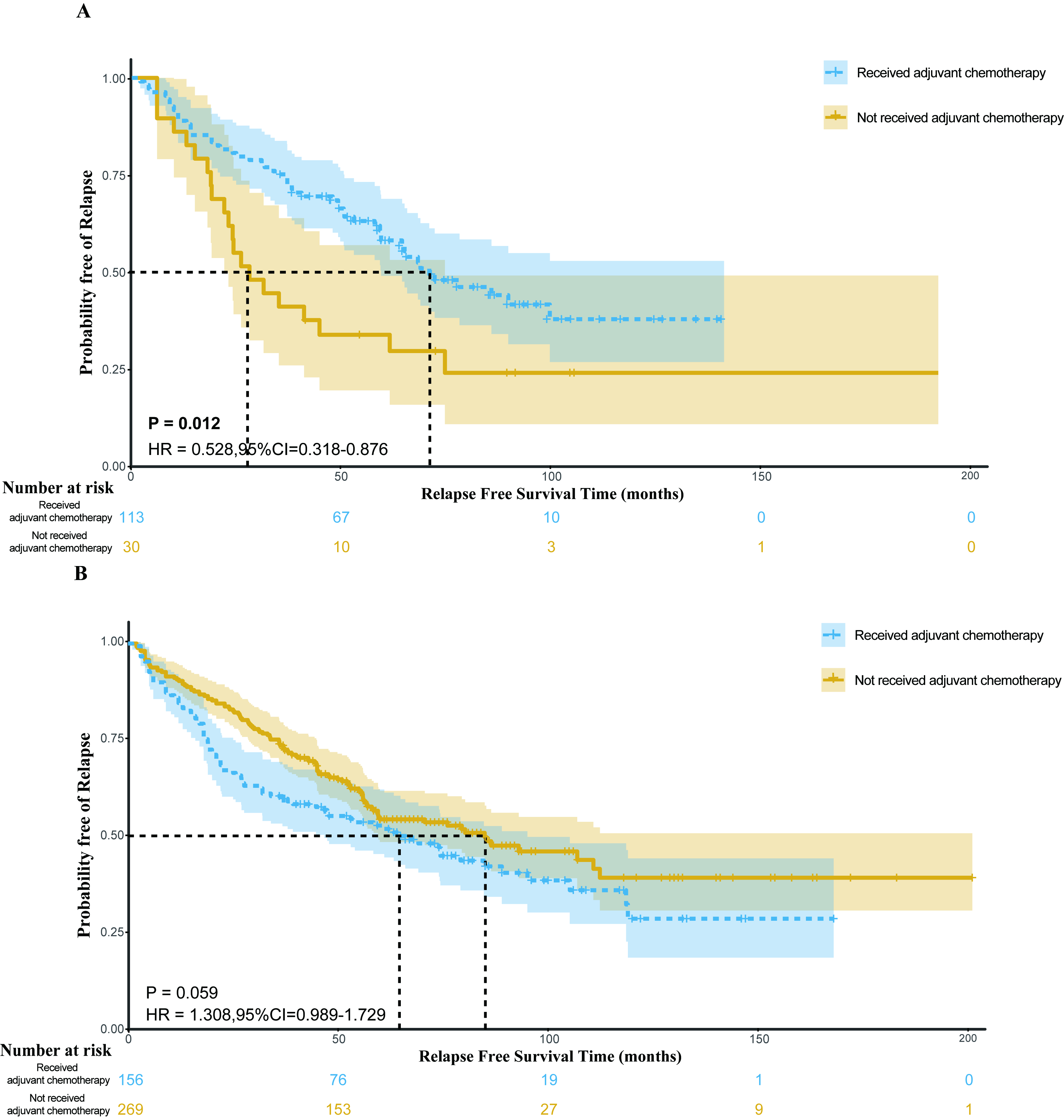

Supplement: Supplementary file 1 — FigS1 [file CAM4-9-3043-s001.tif]

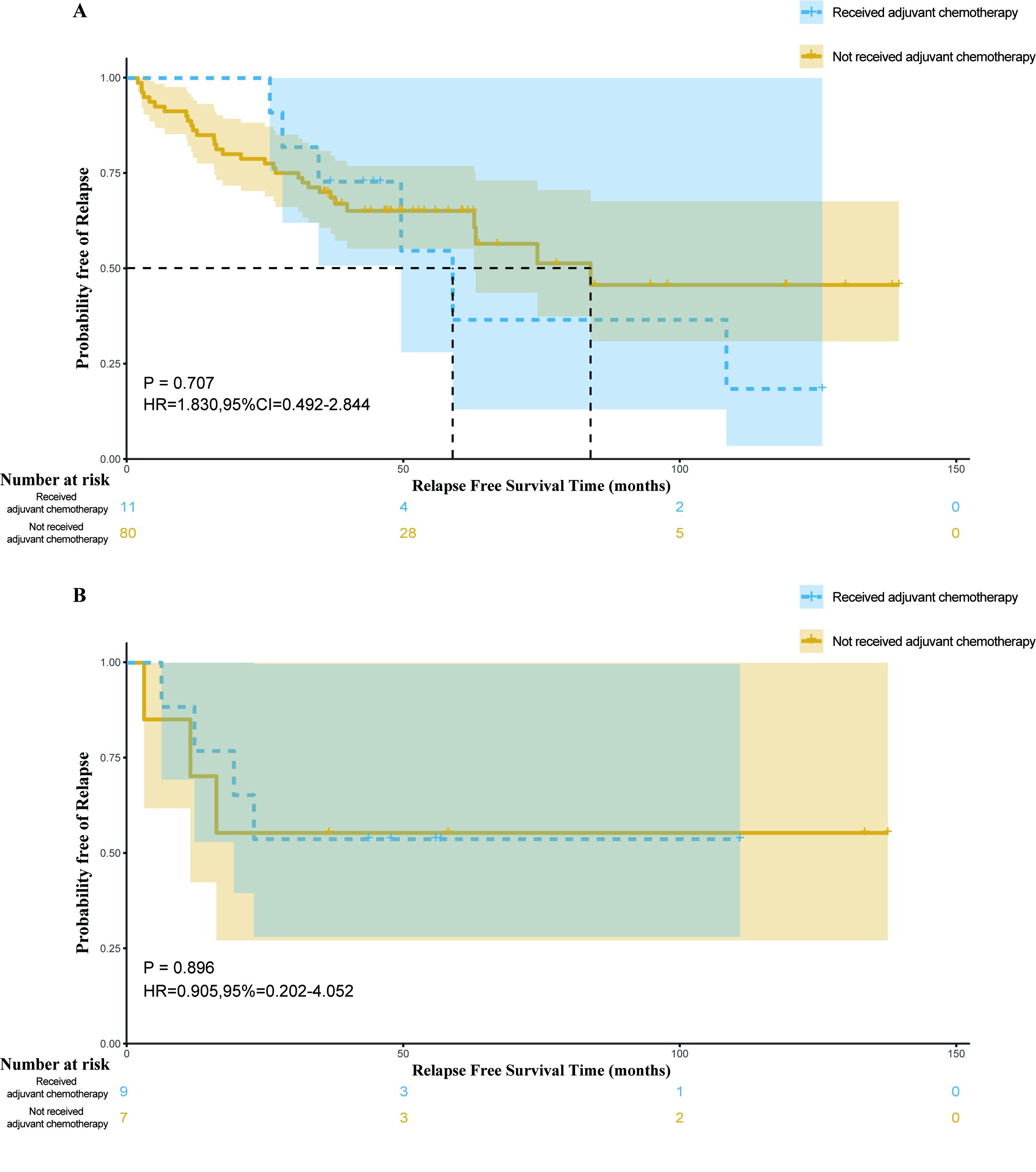

Supplement: Supplementary file 2 — FigS2 [file CAM4-9-3043-s002.tif]

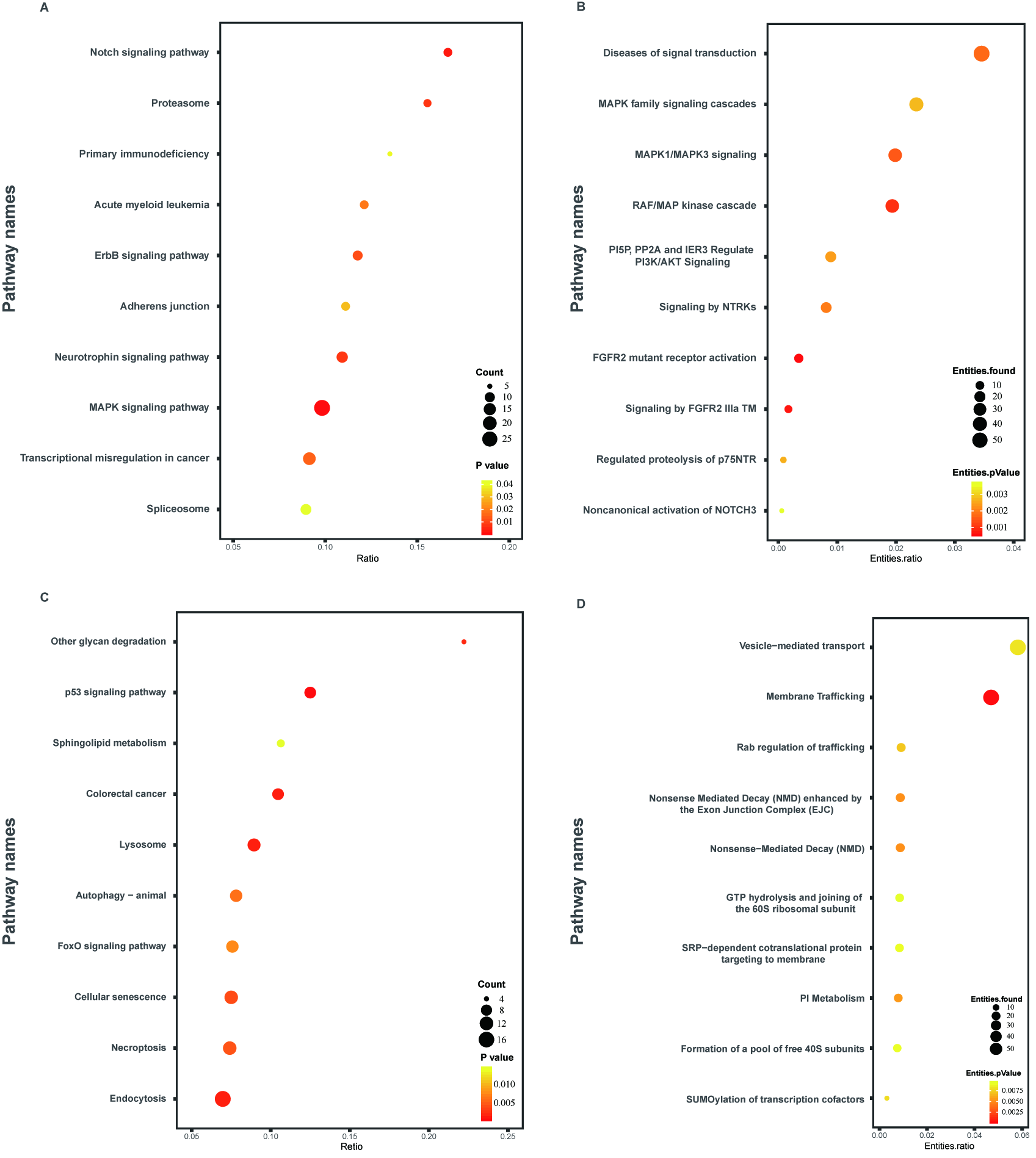

Supplement: Supplementary file 3 — FigS3 [file CAM4-9-3043-s003.tif]

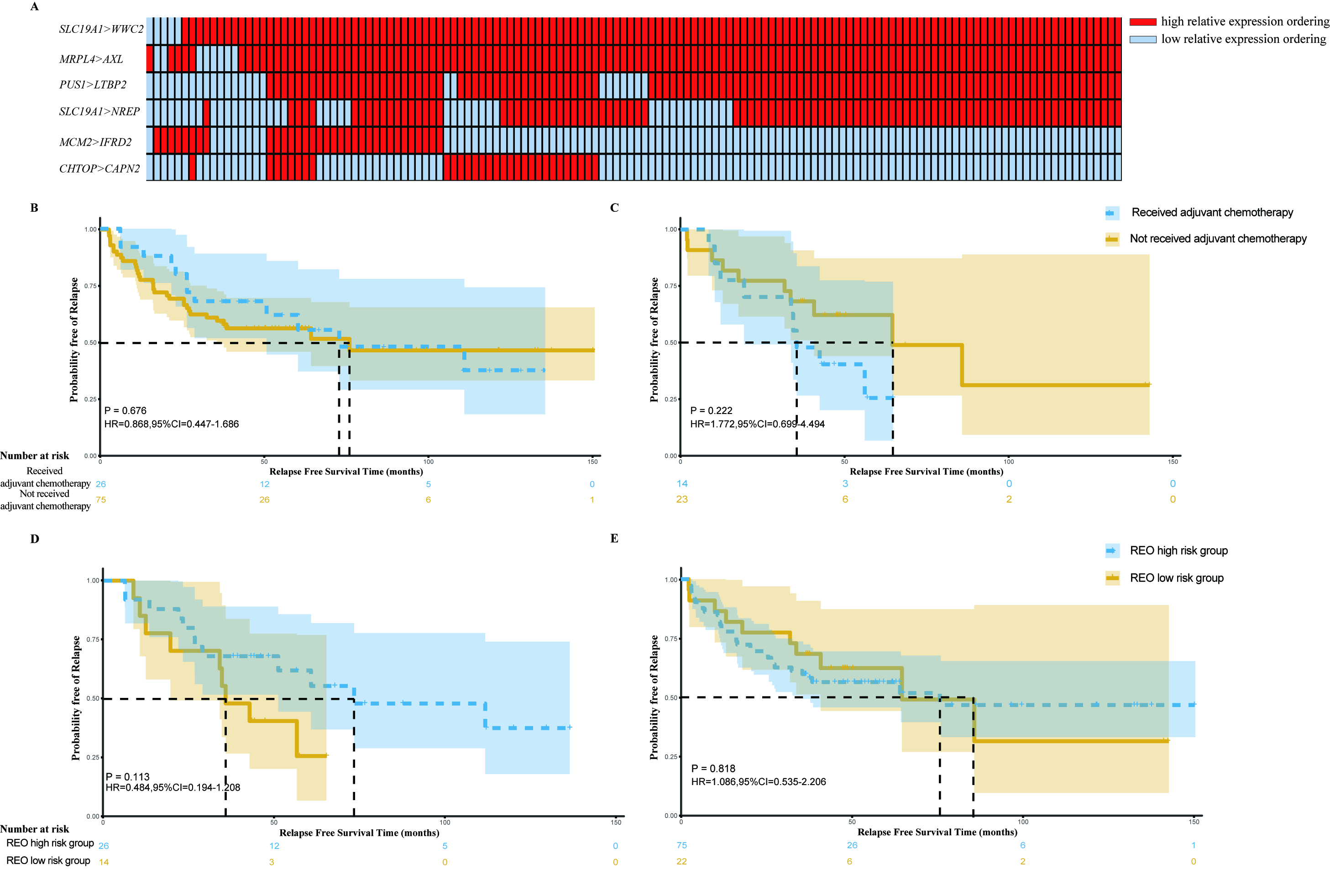

Supplement: Supplementary file 4 — FigS4 [file CAM4-9-3043-s004.tif]
