# Supplementary material for: Predicting the effect of 5‐fluorouracil–based adjuvant chemotherapy on colorectal cancer recurrence: A model using gene expression profiles
Source: Cancer Med. 2020 Mar 9;9(9):3043–56. doi: 10.1002/cam4.2952 (PMC7196071; doi:10.1002/cam4.2952)
Supplement: Supplementary file 5 — TableS1‐S3 [file CAM4-9-3043-s005.docx]

**Supplementary Table 1** Summary of 706 CRC patients analyzed in this study

|  |  | TCGA  [n=138] | GSE14333  [n=145] | GSE29621  [n=31] | GSE39582  [n=392] |
| --- | --- | --- | --- | --- | --- |
| Stage |  |  |  |  |  |
|  | II | 91(22.69%) | 82(20.45%) | 15(3.74%) | 213(53.12%) |
|  | III | 47(15.41%) | 63(20.66%) | 16(5.25%) | 179(58.68%) |
| Adjuvant Chemotherapy | |  |  |  |  |
|  | Not received | 98(24.69%) | 82(20.65%) | 11(2.77%) | 206(51.89%) |
|  | Received | 40(12.94%) | 63(20.39%) | 20(6.48%) | 186(60.19%) |

**Supplementary Table 2** Comparison of relapse-free survival between patients received ACT and those with surgery only in the test cohort.

|  | Subgroup(recurrence/N) | Hazard Ratio (95% CI); P value* | |
| --- | --- | --- | --- |
|  |  | Univariate Analysis | Multivariate Analysis^#^ |
| Age (years) | <70 (35/78) | 1.155(0.594, 2.246); 0.671 | 0.759(0.287,2.004); 0.578 |
|  | ≥70(29/60) | 1.174(0.408,3.378); 0.766 | 0.870(0.274,2.757);0.812 |
| Sex | Male (38/78) | 1.122(0.530,2.374); 0.763 | 0.394(0.127,1.221); 0.107 |
|  | Female (26/60) | 1.181(0.546,2.555); 0.672 | 1.412(0.472,4.223); 0.537 |
| TNM stage | II (37/91) | 1.184(0.493,2.847); 0.705 | 1.378(0.558,3.407); 0.487 |
|  | III (27/47) | 0.543(0.253,1.163); 0.116 | 0.435(0.170,1.109); 0.081 |
| Predictive group | ACT futile (44/107) | 1.211(0.598,2.454); 0.595 | 1.490(0.673,3.298); 0.325 |
|  | ACT benefit (20/31) | 0.345(0.140,0.850); 0.021 | 0.266(0.095,0.742); 0.011 |

^*^ Using samples with surgery only as reference

^#^ Analysis adjusted for Age, Sex, TNM stage and Predictive group, and adjuvant chemotherapy

**Supplementary Table 3** Logistics analysis of clinicopathologic factors associated with ACT in the test cohort.

|  |  | Patients received ACT (N%) | Patients not received ACT (N%) | OR (95% CI) | P^*^ |
| --- | --- | --- | --- | --- | --- |
| Age | |  |  |  | 0.001 |
|  | <55 | 14 (35.00%) | 12 (12.24%) | 1 |  |
|  | 55-65 | 12 (30.00%) | 16 (16.33%) | 0.643(0.219-1.883) |  |
|  | 65-75 | 11 (27.50%) | 38 (38.78%) | 0.248(0.089-0.690) |  |
|  | >75 | 3 (7.50%) | 32 (32.65%) | 0.080(0.020-0.330) |  |
| Race | |  |  |  | 0.086 |
|  | White | 22 (55.00%) | 56 (57.14%) | 1 |  |
|  | Black/African | 7 (17.50%) | 4 (4.08%) | 4.455(1.185-16.738) |  |
|  | Asian | 1 (2.50%) | 2 (2.05%) | 1.273(0.110-14.758) |  |
|  | Unknown | 10 (25.00%) | 36 (36.73%) | 0.707(0.300-1.666) |  |
| Site | |  |  |  | 0.367 |
|  | Colon | 29 (72.50%) | 78 (79.59%) | 1 |  |
|  | Rectum | 11 (27.50%) | 20 (20.41%) | 1.479(0.632-3.462) |  |
| Gender | |  |  |  | 0.005 |
|  | Male | 15 (37.50%) | 63 (64.28%) | 1 |  |
|  | Female | 25 (62.50%) | 35 (35.72%) | 3.000(1.400-6.427) |  |
| Histological Type | |  |  |  | 0.709 |
|  | AC | 36 (90.00%) | 86 (87.76%) | 1 |  |
|  | MC | 4 (10.00%) | 12 (12.24%) | 0.796(0.241-2.635) |  |
| T Stage | |  |  |  | 0.031 |
|  | T1 | 2 (5.00%) | 1 (1.02%) | 1 |  |
|  | T2 | 31 (77.50%) | 92 (93.88%) | 0.168(0.015-1.923) |  |
|  | T3 | 7 (17.50%) | 5 (5.10%) | 0.700(0.049-10.014) |  |
| N Stage | |  |  |  | <0.001 |
|  | N1 | 11 (27.50%) | 80 (81.64%) | 1 |  |
|  | N2 | 17 (42.50%) | 9 (9.18%) | 13.737(4.930-38.276) |  |
|  | N3 | 12 (30.00%) | 9 (9.18%) | 9.697(3.327-28.261) |  |
| TNM Stage | |  |  |  | <0.001 |
|  | II | 11 (27.50%) | 80 (81.64%) | 1 |  |
|  | III | 29 (72.50%) | 18 (18.36%) | 11.717(4.948-27.746) |  |

Abbreviations: OR, odds ratio; CI, confidence interval.

^*^ P values were made by Univariate Logistics Analysis.
